# Age Differences in the Association Between Cardiovascular Disease, Depression, and Suicide Risk

**DOI:** 10.1093/geroni/igab046.3559

**Published:** 2021-12-17

**Authors:** Ruifeng Cui, Alaa Shalaby, Armando Rotondi, Amy Albright, Judith Callan

**Affiliations:** 1 VA Pittsburgh Healthcare System, Pittsburgh, Pennsylvania, United States; 2 VA Maine Healthcare System, Lewiston, Maine, United States

## Abstract

Cardiovascular disease (CVD) is prevalent among older adults aged 60+ (75%). The literature shows a strong bidirectional association between risk for CVD and risk for depression, although there is limited research regarding whether the strength of this association differs by age. CVD may also be related to suicide risk; however, the literature is both limited and mixed, with studies inconsistently finding an association. Additionally, no known studies have investigated age differences in this relationship. The present study examined the association between CVD (assessed via diagnostic checklist), depression (PHQ-8), and suicide risk (SBQ-R), as well as whether these associations differed by age. The current sample consisted of 301 younger adults (aged 18-40) and 432 older adults (aged 60+) recruited online through Mechanical Turk (younger adults: 78.1% white, 46.5% female; older adults 91.4% white, 56.3% female). Older adults had more CVD diagnoses (M=0.9) than younger adults (M=0.3). The association between CVD (i.e., 1+ CVD diagnoses vs. 0 diagnoses) and mental health was moderated by age (depression interaction p<.001; suicide risk interaction p=.033). Among younger adults, presence of CVD diagnosis was associated with 85% higher depression symptoms (M=6.1 vs 11.3) and 48% higher suicide risk scores (M=5.8 vs 8.6) when compared to no diagnoses. CVD had less of a negative impact among older adults and was associated with 64% higher depression symptoms (M=3.1 vs 5.1) and only 14% higher suicide risk scores (M=4.3 vs 4.9). Providers treating CVD may consider assessing and addressing depression and suicide risk, especially among younger patients with CVD.

